# A quantitative investigation of linker histone interactions with nucleosomes and chromatin

**DOI:** 10.1038/srep19122

**Published:** 2016-01-11

**Authors:** Alison E. White, Aaron R. Hieb, Karolin Luger

**Affiliations:** 1 Department of Chemistry and Biochemistry, University of Colorado Boulder, Boulder, CO 80303; 2 Institute for Genome Architecture and Function Colorado State University, Fort Collins CO 80523-1870; 3 Howard Hughes Medical Institute, University of Colorado Boulder, Boulder, CO 80303

## Abstract

Linker histones such as H1 are abundant basic proteins that bind tightly to nucleosomes, thereby acting as key organizers of chromatin structure. The molecular details of linker histone interactions with the nucleosome, and in particular the contributions of linker DNA and of the basic C-terminal tail of H1, are controversial. Here we combine rigorous solution-state binding assays with native gel electrophoresis and Atomic Force Microscopy, to quantify the interaction of H1 with chromatin. We find that H1 binds nucleosomes and nucleosomal arrays with very tight affinity by recognizing a specific DNA geometry minimally consisting of a solitary nucleosome with a single ~18 base pair DNA linker arm. The association of H1 alters the conformation of trinucleosomes so that only one H1 can bind to the two available linker DNA regions. Neither incorporation of the histone variant H2A.Z, nor the presence of neighboring nucleosomes affects H1 affinity. Our data provide a comprehensive thermodynamic framework for this ubiquitous chromatin architectural protein.

Linker histones contribute to chromatin compaction by directly interacting with the nucleosome; they organize linker DNA and thereby promote inter-nucleosome interactions. Linker histones are a family of ubiquitous small proteins that are comprised of a short (~20 amino acid) N-terminal tail, a central winged-helix-like globular domain (~80 amino acids), and a highly positively charged C-terminal domain (CTD, about 100 residues in length). With the exception of yeast, all eukaryotic organisms have multiple isoforms of linker histones with partially redundant functions^1^. Combined, their numbers average to approximately one linker histone per nucleosome. Depletion of linker histones in the cell results in reduced nucleosome repeat length, and in the misregulation of the transcription of many genes^2^. In addition to compacting chromatin, linker histones interact with a surprising number of diverse nuclear proteins, suggesting functions beyond nucleosome interaction^3^.

Linker histones prefer nucleosomal and four-way junction DNA over free DNA^4^,^5^,^6^, suggesting that they recognize a specific DNA geometry. However, despite the fact that the structures of the nucleosome and of the globular domain of linker histone H5 (the avian equivalent of H1.0) have been determined many years prior^7^,^8^, the molecular details of this fundamental chromatin complex are still largely unknown. To date, the most detailed description comes from NMR studies of the globular domain of *Drosophila* H1 in complex with a nucleosome that is centrally positioned on 167 base pairs of DNA^9^. This study proposes a model in which H1 interacts with DNA at the nucleosomal dyad, with additional interactions along ~10 base pairs of one linker DNA, and minor interactions with the second linker arm. NMR data also suggests that the H2A C-terminal tail is in close proximity to the globular domain of H1, although direct interactions have not been demonstrated. This finding (together with previously published results^10^) led to the hypothesis that nucleosomes containing H2A variants might interact differently with linker histone.

Recently, the structure of a 12mer nucleosomal array in complex with full length H1 was determined by cryo-electron microscopy^11^. Although molecular details (especially of H1 regions beyond the globular domain) cannot be discerned at a resolution of 11 Å, the linker histone appears to interact with the nucleosomal dyad and with both linker arms, thereby altering linker conformation and thus fiber geometry. A similar conclusion was reached by a combination of hydroxyl-radical footprinting and modeling, and it was further suggested that a short region of the H1 CTD is responsible for the formation of the characteristic linker stem configuration in trinucleosomes^12^.

A variety of approaches using different histone H1 variants support other binding models (summarized in^13^). Linker histones protect an additional 20 base pairs (or a total of 168 base pairs) of DNA from nuclease digestion^14^, but it is controversial whether this protection occurs on one or on both linker DNA arms extending from the nucleosome core particle (e.g.^12^,^15^,^16^,^17^,^18^,^19^). The literature is complicated by the fact that different linker histone isoforms might exhibit different interactions with nucleosomes. Earlier nucleosome preparations did not have the exquisite positioning properties observed by the ‘601’ sequence^20^, and thus the role of linker DNA extensions was difficult to assess.

A valid method to determine the ‘necessary and sufficient’ contributions of linker DNA and of H1 tail domains to complex formation is to measure the binding affinities of nucleosomes with various linker lengths to different linker histone constructs. Gel shift experiments were recently used to determine a minimal length requirement of 10 base pairs of symmetrically extending linker DNA, although interactions with at least 21 base pairs from the edge of the nucleosome core were noted^21^. Historically, a wide range of affinities has been reported for various versions of linker histone-nucleosome complexes, using different *in vitro* and *in vivo* approaches. Quantifying nucleosome gel shifts upon H1 binding, using radiolabeled nucleosomes, typically gives affinities between 1–10 nM^6^,^21^,^22^. More recently, affinities of about 300 nM were reported from isothermal calorimetry (ITC)^9^. Using a fluorescence quenching assay, we have recently published values around 1 nM^23^. Quantitative information on H1 interactions with chromatin *in vivo* have also been obtained from FRAP (Fluorescent recovery after photo-bleaching) experiments^17^,^24^,^25^,^26^. Even though equilibrium binding constants cannot be derived, this method was particularly useful in dissecting the contributions of the C-terminal domain of H1.

The positively charged, intrinsically disordered H1 CTD contributes to the interaction by organizing additional linker DNA and neutralizing its charges (reviewed in^27^). The sequence of the CTD is quite variable between H1 species and isoforms, and amino acid composition rather than the precise sequence is the major determinant of CTD function^28^. Interaction with the nucleosome induces α-helical structures within the conserved S/TPKK domains of the CTD^27^,^29^,^30^. Surprisingly, it was recently shown that deletion of the entire CTD does not contribute significantly to the binding affinity to mono-nucleosomes *in vitro*^21^, whereas it appears to be important in a more complex cellular environment^17^,^24^,^25^.

Here, we use a solution-state fluorescence assay (HI-FI FRET^23^) to dissect the contributions of the two nucleosomal linker DNA extensions and of the H1 CTD to complex formation. Using defined mono- and trinucleosome substrates, we characterized the interactions of H1 with nucleosomes and visualized the effect of the H1 interaction on the relative arrangement of nucleosomes within an array. We find that only one linker arm primarily contributes to H1 specificity for the nucleosome. The replacement of histone H2A with H2A.Z does not affect H1 interactions with the nucleosome. Together, our data suggest a refined model for the H1-nucleosome complex in a native chromatin context.

## Results

### H1 recognizes nucleosomal DNA geometry

**Figure 1:**
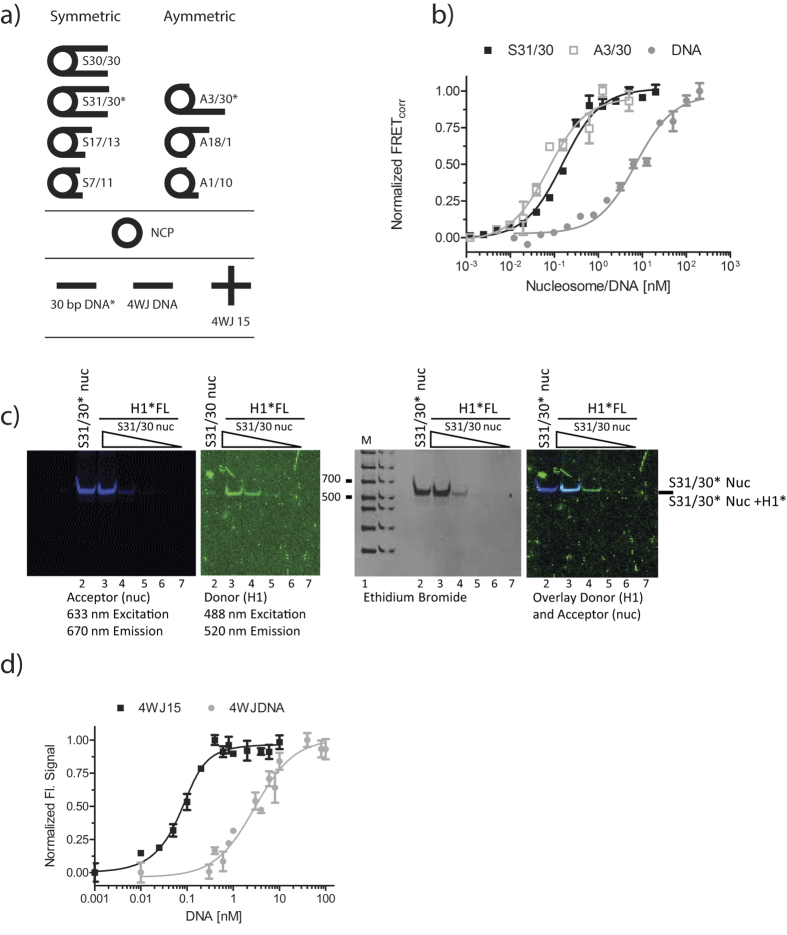
H1 recognizes nucleosomal linker geometry. (**a**) Schematic of DNA and mono-nucleosome substrates used in this study. Nucleosomes with symmetric and asymmetrically extending linker arms are designated with the prefix S and A, respectively. 5′ end of 601 DNA is on top. Fluorescently labeled arms are indicated*. NCP is nucleosome core particle with 147 bp DNA (devoid of linker DNA). 30 bp DNA has the same sequence as the 30 bp DNA linker in nucleosomes; 4WJDNA is a 30 bp DNA fragment that forms a four-way junction with 15 bp arms (4WJ15). (**b**) Representative FRET binding curves. H1_FL_ was kept constant at 0.5 nM, while nucleosome or DNA was titrated (0–20 nM). The normalized data was fit with a quadratic equation (*Eq.*3) for S31/30 (■) and A3/30 (□) nucleosome. For measurements with linear DNA, 1 nM H1_FL_ was used, and the data was fit with a one site binding equation (*Eq. 2*). (**c**) 5% native PAGE of samples taken from microplate of S31/30 FRET assay shown in b). The gel was visualized at the indicated wavelengths, and then stained with ethidium bromide. Lane 1: marker (Biorad 50–2kb); lane 2: 20 nM S31/30 nucleosome without H1; lanes 3–7: 0.5 nM H1 with decreasing amounts of S31/30 nucleosome (20, 2.5, 0.3125, 0.039, 0.0049 nM, respectively). **(d**) Representative (de)quenching curves of four-way junction and linear DNA. (De)quenching assays were completed at 20 mM KCl where H1_FL_ was at 0.5 nM and 4WJ was titrated (0–100 nM); curves were fit to a quadratic (*Eq.*3) or one-site binding equation (*Eq.* 2). All measured affinities including errors are listed in Table 1.

To quantify the preference of H1 for particular DNA configurations, we used a solution-state assay based on fluorescence resonance energy transfer (HI-FI FRET) to compare interactions between H1 and nucleosomes with one and two linker DNA extensions, with free DNA, and with a DNA construct that forms a four-way junction (Fig. 1a).

We first measured the binding affinity of full length H1 (H1_FL_) for a nucleosome with two ~30 base pair linker DNA extensions (S31/30 nucleosomes, Fig. 1a). Nucleosomal DNA was labeled with Atto 647N eleven base pairs from one end, to ensure efficient FRET to a labeled linker histone bound near the nucleosomal dyad axis (<100 Å distance), and nucleosomes were titrated into a constant amount of Oregon-green labeled linker histone. A representative binding curve for H1 to S31/30 nucleosome is shown in Fig. 1b, demonstrating an exceedingly tight interaction, with a K_d_ of 0.047 nM (Table 1). This value is significantly lower than previously published values (see discussion). To verify that the H1-nucleosome complex remains intact during the experiment, samples were removed from the microplate after scanning and analyzed by native PAGE (Fig. 1c). Fluorescence signals for H1 and the nucleosome co-localize throughout the titration, and no change in nucleosome migration was observed upon H1 binding. No free DNA was released in the course of the titration, indicating that nucleosomes do not dissociate during the experiment. This demonstrates that our experiments indeed measure the interaction of H1 with an intact nucleosome.

To test whether one or both linker DNA regions engage with H1, we prepared a nucleosome construct with one 30 bp linker DNA segment, while the other end of the DNA extends only three base pairs beyond the histone octamer (A3/30 nucleosomes; Fig. 1a). These asymmetric particles bind H1_FL_ with only six-fold weaker affinity compared to S30/30 nucleosomes (Fig. 1b). This suggests a binding mode in which full length H1 predominantly associates with one linker arm and with DNA near the nucleosomal dyad.

The affinity of H1_FL_ for a 30 bp linear DNA fragment with the same sequence (and identical labeling position) as one of the two nucleosome linker arms was determined under identical conditions (Fig. 1b), resulting in a 60- 150-fold weaker K_d_ compared to both nucleosomes. We also measured the affinity of H1_FL_ for a four way junction DNA, which is constructed of four symmetric 15 bp linker arms (4WJ15; Fig. 1a)^31^. A linear 30 bp DNA fragment of the same sequence served as a control (Fig. 1d). H1 binds 4WJ15 with 100-fold tighter affinity than its linear equivalent 4WJDNA (0.035 nM and 3.75 nM, respectively), indicating that a DNA architecture where DNA segments are oriented at an angle is specifically recognized by H1 and is required for high-affinity interactions. Because these latter measurements were performed at lower ionic strength (20 mM KCl) to maintain the four-way junction, they are not directly comparable to data obtained with nucleosomes at 150 mM KCl.

### H1 requires greater than 11 bp of a single linker DNA for high affinity binding

The minimal length requirement for symmetrically extending linker DNA was previously reported to be 10/10 base pairs^21^. However, since our results suggest asymmetric binding of H1 to nucleosomes, we also wanted to test the length requirements for asymmetrically extending linker DNA. We prepared nucleosomes with varying linker lengths (Fig. 1a), and compared their ability to compete for FRET between H1_FL_ and S31/30 nucleosomes by monitoring loss of FRET upon addition of unlabeled competitor nucleosome. This eliminates the necessity to fluorescently label each nucleosome sample. We chose this approach to avoid introducing an additional variable by changing fluorophore location; furthermore we were concerned that labeling nucleosome substrates with little to no linker DNA might impair DNA wrapping around the octamer, thereby affecting H1 interactions. The half maximal inhibitory concentration (IC_50_: *eq.* 4) obtained from such competition experiments can be converted into an apparent dissociation constant (*eq.* 5).

**Figure 2:**
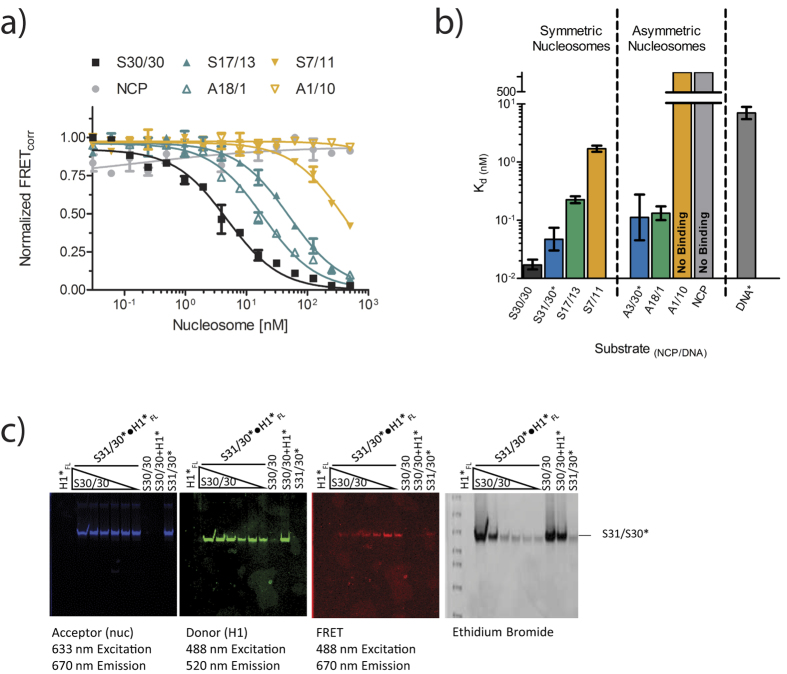
H1_FL_ requires >11 bp of DNA and binds nucleosomes asymmetrically. **(a**) Representative competition curves fit with an IC_50_ curve (*eq.* 4). Unlabeled S30/30 nucleosome (■) competes labeled H1_FL_ from labeled S31/30 nucleosome. Using *eq.* 5, the IC_50_ value was converted to a K_d_. Other competing nucleosomes were tested as indicated. Inability to compete indicates no binding of the competitor. **(b**) The K_d_ values from a) are shown in a logarithmic bar graph. *denotes a direct FRET measurement. The error bars are 95% confidence intervals. All measured affinities are summarized in Table 1. **(c**) 5% native PAGE of samples taken from the microplate competition assay with S31/30. Nucleosome assembled on Atto647N labeled DNA (acceptor) were kept constant at 10 nM and pre-incubated with 1.0 nM Oregon green labeled H1_FL_ (donor), unlabeled S30/30 nucleosome was then titrated (0nM-500nM). The same gel was visualized at the indicated wavelengths, then stained with ethidium bromide. Lane 1: DNA size marker (Biorad 50–2kb); lane 2: H1_FL_ alone (does not enter the gel); lane 3–8: S31/31●H1 complex incubated with decreasing amounts of unlabeled S30/30 nuc (500, 62.5, 15.6, 1.95, 0.244, 0.0305 nM respectively); lane 9: 500 nM S30/30; lane 10: 500 nM S30/30 with 1 nM H1_FL_, lane 11: 20 nM S31/30 alone. Fluorescently labeled species are indicated with an asterisk.

To validate the competition approach, we first tested competition by S30/30 nucleosomes (Fig. 2a). This nucleosome is nearly identical to S31/30 nucleosomes, differing by only one base pair and lacking the fluorophore. From an IC_50_ value of 3.7 nM we extract an apparent K_d_ of 0.02 nM, which is within error of the K_d_ obtained by direct FRET measurements (Fig. 2b; Table 1). To confirm competitive binding, samples were removed from the microplate and subsequently analyzed by native PAGE (Fig. 2c). H1 and S31/30 nucleosomes co-migrate, as seen by the presence of FRET signal in the gel. Upon addition of unlabeled competitor S30/30 nucleosomes, FRET signal was no longer observed, indicating that the interaction was lost. Both acceptor and donor signals remained constant, demonstrating that H1 remains consistently bound to a nucleosome substrate (switching binding partner from S31/30 to S30/30), and that S31/30 nucleosomes remain intact throughout the experiment. Unbound H1 does not enter the gel. Together, this establishes competition as a valid approach for determining the affinity of H1 for multiple nucleosome substrates. Similar agreement between quenching, FRET, and competition was observed for six additional H1-nucleosome pairings that varied in K_d_ over a factor of 2000 (Table 1).

To correlate our findings with previously published results^21^, we next examined the effect of shortening the length of linker DNA extending symmetrically on both sides (Fig. 1a). Competition curves are shown in Fig. 2a (solid symbols), and affinities derived from these and direct FRET measurements are summarized in Fig. 2b and Table 1. In summary, S31/30 and S30/30 nucleosomes exhibit the strongest affinity for H1, while a reduction in linker length by ~15 base pairs (S17/13 nucleosomes) results in only 5-fold weaker affinity (0.23 nM). Removal of an additional 6 bp (S7/11 nucleosomes) reduces the affinity by a factor of 35 compared to S31/30 nucleosomes (1.73 nM). A nucleosome core particle (NCP) completely lacking linker DNA does not compete for H1_FL_, consistent with published results^21^. However, unlike these previous experiments, HI-FI FRET detects sub-nanomolar affinities in solution, and reveals a cumulative effect of linker DNA length beyond 11 base pairs to overall H1 affinity.

We next tested nucleosome constructs with only one extending linker arm (A3/30, A18/1, and A1/10). Compared to A3/30, H1 affinity was unaffected by deleting 12 base pairs from the remaining linker arm (A18/1). No competition (that is, no binding) was observed when 20 base pairs were deleted (A1/10; Fig. 2a,b, supplementary Figure 1a). This suggests that the majority of linker histone H1 interactions occur with DNA at the nucleosomal dyad, and with one DNA linker arm extending more than 10, but no more than 18 base pairs from the nucleosome. Our data demonstrate that minor contributions are made by the second linker arm. This is consistent with a protection of ~160 base pairs of nucleosomal DNA in the presence of H1^14^, in an asymmetric binding mode.

#### Neighboring nucleosomes do not contribute to H1 binding

**Figure 3:**
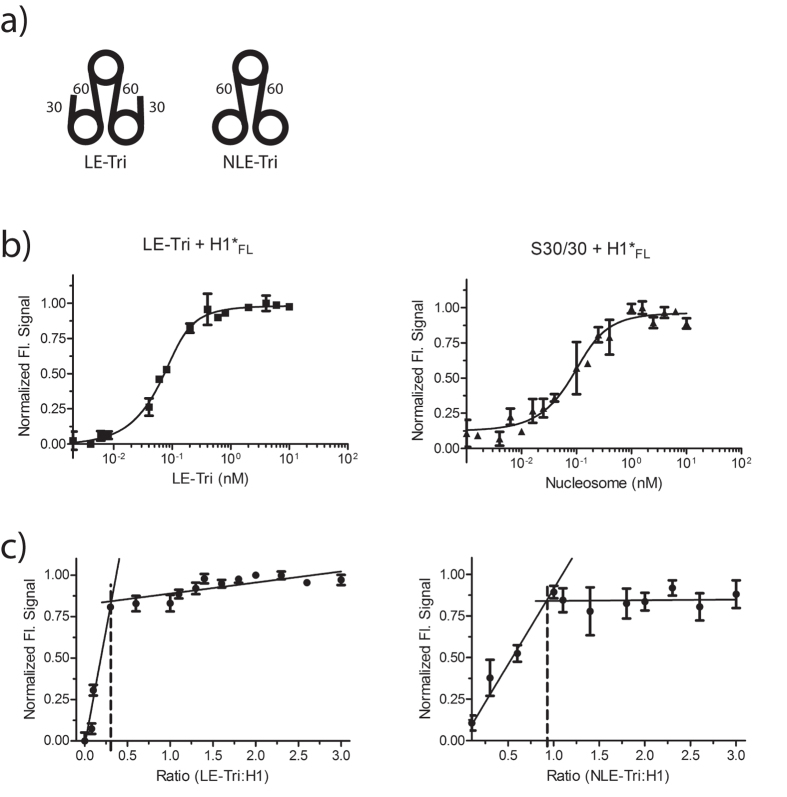
Neighboring nucleosomes do not contribute to H1 binding. **(a**) Schematic of trinucleosome constructs. The constructs differ only in the amount of DNA extending from the two terminal nucleosomes. **(b**) Representative (de)quenching curve of LE-Tri, and S30/30 with H1_FL_. H1 was held constant at 0.1 nM and trinucleosome was titrated (0–20 nM respectively); curves were fit with a quadratic equation (*Eq.* 3). K_d_ values obtained for S30/30 are identical within error of values obtained by FRET (Table 1). **(c**) Stoichiometry of H1_FL_ complexes with trinucleosomes (LE-Tri; left, and NLE-Tri; right). Trinucleosomes were titrated (0.8–30nM) into a constant amount of H1 (10nM). For LE-Tri, we find a molar ratio of 0.3 LE-Tri to one H1 (or 1 H1 per nucleosome). For NLE-Tri we observe a stoichiometry of ~0.9 NLE-Tri per H1 (or one H1 per trimer).

We next wanted to test whether H1 interacts with more than one single nucleosome in an array. To this end, we tested trinucleosome substrates previously designed to characterize nucleosome – PARP-1 interactions^32^. In these constructs, three nucleosomes are connected by 60 bp of linker DNA, to recapitulate a 207 bp nucleosome repeat length. Although nucleosome repeat length varies considerably between organisms and cell types, and is affected by the presence of linker histones^2^, a 207 bp repeat length is commonly used for *in vitro* studies (e.g.^28^). The two types of trinucleosomes assayed here differ in the presence/absence of 30 bp linker ends extending from the two terminal nucleosomes (Fig. 3a; Linker-Ended trinucleosomes, or LE-Tri; and Non-Linker-Ended trinucleosomes, or NLE-Tri). Using HI-FI fluorescence (de)quenching, we found that both substrates bind H1_FL_ with the same high affinity as S30/30 mono-nucleosomes (Fig. 3b, Table 1). As a control, we tested S30/30 nucleosome by (de)quenching, and found its affinity for H1_FL_ to be within error of that measured by FRET (Fig. 1b; 0.039 nM), confirming HI-FI FRET and fluorescence (de)quenching as valid approaches for measuring affinities.

We recently determined that one H1_FL_ molecule binds per mono-nucleosome with symmetrically extending linker DNA (S30/30; reference^23^). Given the asymmetric linker DNA requirements for H1 binding determined above, we predicted that one linker histone would bind per nucleosome in both NLE and LE-trinucleosomes. To test this, stoichiometric measurements were performed by titrating trinucleosomes into a constant concentration of labeled H1, and followed fluorescence (de)quenching throughout the titration series. We find that indeed three H1 molecules bind to LE-Tri, whereas only one H1 binds to NLE-Tri (Fig. 3c). This suggests that H1 binds to the nucleosomal dyad / linker DNA and alters the configuration of one or both linker arms in a manner that precludes binding of a second H1 to the either one of the two terminal nucleosomes.

#### H1 influences the geometry of tri-nucleosomes

**Figure 4:**
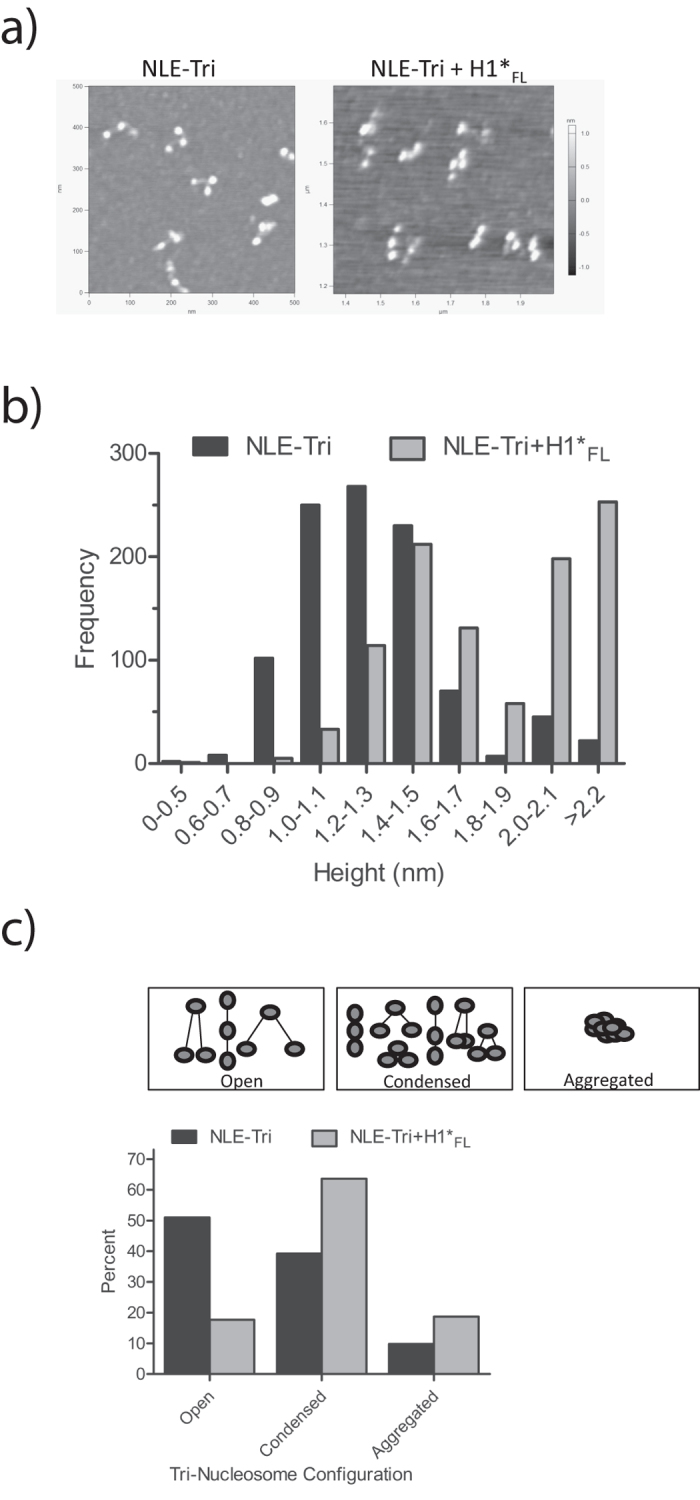
H1 compacts trinucleosomes. **(a**) NLE-Tri was imaged by atomic force microscopy (AFM) in absence of H1_FL_ (left), or with a 1:1 ratio of H1 per tri-nucleosome (right). **(b**) Binned height profiles of both NLE-Tri alone and in the presence of H1_FL_. A minimum of 9 separate images were used to complete height traces (Fig. S4A) on a total of 1005 nucleosomes either with or without H1_FL_ and those heights are depicted in this graph. The mean height distribution of NLE-Tri alone is ~1.2 nm and increases to ~1.5 nm in the presence of H1_FL_. Importantly, the propensity for aggregates increases significantly with H1_FL_ present. **(c**) Each of the observed arrangements was seen in an open conformation (longer distance between the nucleosomes - open), or in a condensed conformation with closely spaced nucleosomes. Aggregation of sample was also observed. A minimum of seven separate images for both NLE alone or with H1_FL_ were used to count the number of trinucleosomes in each group for a total of 481 NLE-tri alone and 524 NLE-Tri with H1_FL._ The graph depicts the number of trinucleosomes found in each group in the absence or presence of H1_FL_.

To investigate the structural effect of H1 on trinucleosomal arrays, we visualized NLE-Tri in absence and presence of H1_FL_ by Atomic Force Microscopy (AFM; Fig. 4a, and supplementary Figure 2). A minimum of nine images were used to measure the height profiles of 1005 particles in absence of H1_FL_, and an average particle height of 1.3 nm was determined (Fig 4b). H1_FL_ and NLE-Tri were combined at the previously determined stoichiometry of one H1_FL_ per NLE-Tri, and imaged under identical conditions. The average height profiles derived from 1005 nucleosomes increased from 1.3 to ~1.5 nm, and a number of particles were above 2 nm in height.

The most frequent arrangement of trinucleosomes in absence of linker histones is triangular, but several other arrangements were also observed (e.g. condensed or linear arrangement (Fig. 4c). Similar types of arrangements were also seen in the presence of H1_FL_, but with smaller distances between individual nucleosomes within a trinucleosome. Without H1, 50% of particles were in an open configuration, while this number drops to 18% in presence of H1. Conversely, in absence of H1, 39% of the trinucleosomes were in a condensed configuration, while in the presence of H1 this number increases to 64%. Additionally, the propensity of trinucleosomes to form larger assemblies nearly doubles in the presence of H1_FL_. This data is consistent with the interpretation that H1 affects the geometry of linker DNA, thereby compacting nucleosomal arrays. H1 induced condensation appears to promote self-association between individual nucleosomal arrays.

#### The presence of histone variant H2A.Z in nucleosomes and nucleosomal arrays does not affect the interaction with linker histone

It was shown previously that the replacement of several amino acids in the C-terminal tail of canonical H2A with equivalent residues of the histone variant H2A.Z abolishes the binding of a truncated version of H1 to such nucleosomes^9^. To test this in a more native context, we measured the interaction of H1_FL_ with H2A.Z-containing S30/30 mono-nucleosomes and with H2A.Z-containing NLE-trinucleosomes, using fluorescence (de)quenching (Supplementary Figure 1b,c). We find that both substrates bind H1 with affinities that are within error of those determined for the same substrates containing major-type H2A (Table 1). Importantly, our data for S30/30-H2A.Z obtained through fluorescence (de)quenching confirm the high affinities measured by direct FRET and by FRET competition assays.

#### The C-terminal tail of histone H1 contributes mostly to linker DNA binding in a mono-nucleosome

Two discrete regions within the CTD of H1.0 are responsible for promoting the folding of nucleosome arrays (amino acids 97–121 and 145–169)^9^,^25^,^33^. The contributions of H1 tail regions to the interaction with mono-nucleosomes with symmetrically extending linker DNA arms were recently dissected, showing no positive, and in some cases even inhibitory effects of the CTD^21^.

**Figure 5:**
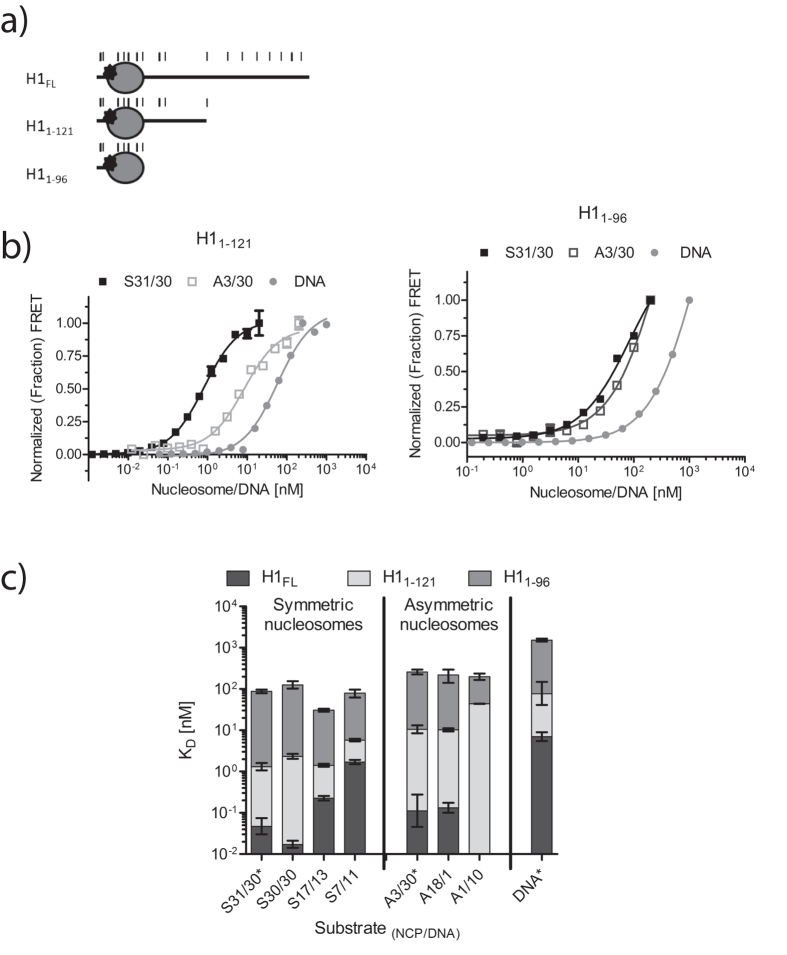
C-terminal tail of H1 contributes mostly to linker DNA binding. **(a**) H1 constructs used; *indicates Oregon Green label at S18C. Five lysine residues are indicated by each thin black line. Each thick black line represents one arginine residue. **(b**) Representative FRET binding curves for H1_1–121_ (left) and H1_1–96_ (right). H1 (donor) was kept at constant concentration of 0.5 nM (5 nM with 30 bp linear DNA) and labeled nucleosome (acceptor) was titrated (0–20 nM: 0–200 for 30 bp linear DNA respectively). Binding isotherms are shown for S31/30, A3/30, and 30 bp DNA. K_d_ values and errors are listed in Table 1. **(c**) Bar graph on logarithmic scale of the average of each replicate K_d_ with 95% confidence interval for each nucleosome or free DNA in this study bound with H1_FL_ (dark gray), H1_1–121_ (light gray), or H1_1–96_ (medium gray). Corresponding binding curves are shown in Figs 1b, 2a and 5b and Supplementary Figure 3a,b.

To further refine the H1 C-terminal tail interactions with nucleosome linker DNA, we measured the affinity of two H1 deletion mutants (H1_1–121_ and H1_1–96_; Fig. 5a) for all nucleosome substrates introduced above. We first compared the affinity of H1_1–121_ and H1_1–96_ for nucleosomes S31/30, A3/30, and free DNA using HI-FI FRET (Fig. 5b,c; Table 1). The basic C-terminal tail significantly contributes to the interaction with nucleosomes. Partial deletion of the H1 tail reduces affinity by a factor of 20–100, and complete deletion results in an 1800–2200-fold reduction in affinity for nucleosomes with two or one linker, respectively. In contrast, the affinity for linear DNA is only reduced by 10 and 200-fold, respectively.

Using competition experiments, we further analyzed the effect of DNA linker length on the affinity for both H1 tail deletion constructs (Supplementary Figure 3a, b). These experiments reveal a trend: the shorter the linker DNA length, the smaller the effect of the CTD deletion on nucleosome binding (Table 1). For example, as pointed out above, H1_FL_ binds S30/30 with 0.02 nM affinity, and successive shortening of the DNA arms results in 10 and 100-fold reduction in binding, respectively. In contrast, the affinity of H1_1–121_ for all mono-nucleosomes with symmetric DNA is similar (1–3 nM). Affinities of H1_1–121_ for asymmetric nucleosomes are overall ~10-fold weaker than those measured for symmetric nucleosomes (8–44 nM, Table 1). A similar trend is observed for H1_1–96_, which binds symmetric nucleosomes with affinities between 30–120 nM, and asymmetric nucleosomes with affinities ranging from 140–250 nM.

For some nucleosome substrates with short linker DNA, H1 tail deletion derivatives exhibit higher affinity than full length linker histones. The most dramatic example is the A1/10 nucleosome, which exhibits no measurable interaction with full length H1, but binds the two shorter H1 variants with affinities of 44 and 140 nM, respectively (Table 1).

Finally, we tested the interaction of H1_1–121_ with trinucleosomes. Surprisingly, we observed no significant loss in affinity compared to full length H1 (supplementary Figure 3c, d; Table 1). It appears that this portion of the H1 CTD does not contribute significantly to the interaction in this context. This is observed for both NLE- and LE trinucleosomes, suggesting that H1 interactions with mono-nucleosomes do not fully recapitulate its interaction with nucleosomal arrays.

## Discussion

Knowledge of the precise binding constants for linker histone H1 interactions with chromatin and DNA is essential to understand its function and dynamic behavior *in vivo*. However, obtaining accurate data has proven difficult due to the propensity of these highly positively charged molecules to bind their substrates non-specifically *in vitro*, resulting in sample precipitation. Furthermore, its strong affinity puts it at the limit of detection for approaches commonly used to obtain binding isotherms. Here, we have optimized several variations of the HI-FI assay to study H1-chromatin interactions in solution, and reproducibly obtained sub-nanomolar affinities of H1.0 derivatives for nucleosomes with different linker DNA geometries. The power of our approach lies in the combination of solution-state FRET with an independent verification of complex formation by native PAGE. Our results shed light on the contributions of linker DNA geometry and the H1 C-terminal basic domain to chromatin interactions.

Overall, the affinities of H1 for nucleosomes reported here suggest much tighter binding than previously reported by others and us. Values between 1 nM, 5–10 nM, and 300 nM have been reported^9^,^21^,^23^. Our own published values of ~1 nM^23^ were obtained by fluorescence (de)quenching in solution, obtained from a nucleosome titration range of 0.05–20 nM. Upon expanding the titration range to even lower nucleosome concentrations, we observed biphasic behavior, indicative of an additional, tighter binding event that was more prominent with fresh H1 preparations (Supplementary Figure 4a, upper panel), but was less apparent with older H1 preparations that exhibited some proteolytic degradation (Supplementary Figure 4b). Deconvolution of the two phases gave rise to a K_d_ of 0.022 and 3.3 nM, respectively (Supplementary Figure 4a, lower panel). In light of the prominent contribution of the CTD to H1 binding demonstrated here, we conclude that the K_d_ values reported earlier were the combined result of proteolytic degradation of H1 and detection limits, likely due to experimental design. Indeed, repeated (de)quenching experiments with an improved H1 preparation devoid of proteolytic degradation resulted in sub-nanomolar affinities for trinucleosomes and for canonical mono-nucleosomes, as well as for H2A.Z-nucleosomes. Discrepancies with other published work^21^ could be due to the fact that gel shift experiments do not allow detection of the exceedingly tight interactions observed in solution, and might be impacted by dissociation kinetics. While the two studies agree on the same minimal length requirements (11 bp) for stable H1 binding, here we also identify significant contributions of linker DNA length beyond the minimal 11 bp length (with affinities ranging from 0.02 to 0.2 to 1.7 nM for nucleosomes with DNA linkers extending 30, 17, and 11 base pairs, respectively). In contrast, Caterino and colleagues report near-identical binding affinities between 4 and 10 nM for similar constructs^21^. Finally, Zhou *et al.* measured affinities of ~500 nM by isothermal calorimetry, using a 10-fold excess of H1 over nucleosomes or DNA^9^. It is possible that the recorded heat changes were due to aggregation, and not due to specific binding.

We show that H1 prefers nucleosomal DNA over free DNA by a factor of ~150. Other studies report only a 2–10 fold difference between free DNA and nucleosomes^22^,^34^, but these have been measured with much longer DNA fragments that allow multiple binding events. Moreover, most studies use H1 titrations, under conditions where H1 can bridge DNA molecules^35^,^36^ which may lead to aggregation. To overcome this, we limited the number of H1 molecules that interact with DNA by using a short 30 base pair DNA fragment. By titrating DNA into limiting amounts of H1, we determined the affinity of a single H1 for the equivalent of an ‘unattached’ DNA linker arm. Because we used the same sequence (and label) for our nucleosome constructs, we can directly compare the contributions of linker DNA inside and outside of context of the nucleosome.

**Figure 6:**
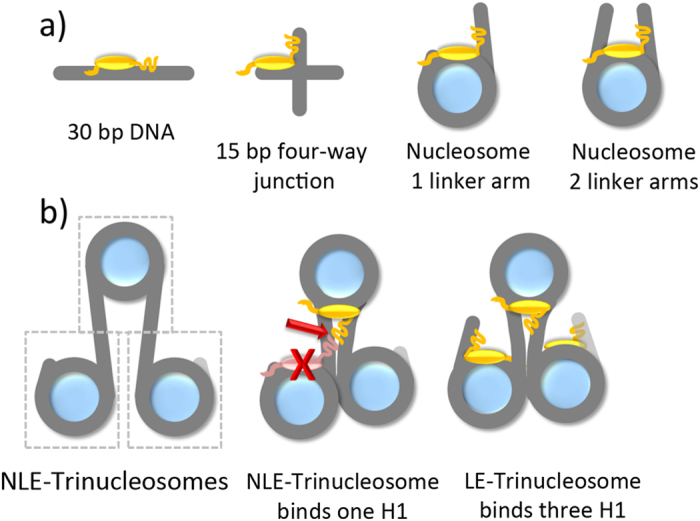
H1 interactions with chromatin. **(a**) H1 (yellow) prefers cruciform and nucleosomal DNA over linear DNA; a single linker DNA arm of ~18 base pairs is sufficient for high affinity binding. **(b**) NLE-Tri nucleosomes have three equivalent binding sites for asymmetric H1 interaction (boxed). H1 compacts trinucleosomes by rearranging linker DNA between nucleosomes, resulting in a 1:1 stoichiometry for NLE-trinucleosomes (middle panel); a second and third H1 molecule is precluded from binding. LE-trinucleosomes accommodate three H1 molecules. Our data suggests that only one linker histone contributes to each linker DNA stem.

The precise location of linker histone on the nucleosomes remains controversial (see Ref. 13 for a recent review of proposed binding modes). For full length H1, we observe the highest affinity for a nucleosome with two 30 base pair linker arms. Deleting one of the two linker arms, and shortening the remaining arm to 18 base pairs reduces the affinity by a factor of 7.6, while further reducing the single linker arm to 10 bp, or complete deletion of both linker arms, completely abolishes binding (Table 1, compare values for S30/30, A1/18, A1/10, and NCP). A nucleosome with 7/11 base pairs of linker DNA binds with 100-fold reduced affinity compared to S30/30 nucleosomes; it is the weakest of all nucleosome constructs for which binding curves were obtained. Our data supports a model in which full length H1.0 binds the nucleosome by interacting with DNA near the nucleosomal dyad and with one linker DNA segment that is between 11 and 18 base pairs in length, bound predominately by the CTD. Minor contributions come from the second DNA linker arm (Fig. 6a), as proposed earlier^9^,^37^, and these interactions are likely facilitated by the second DNA binding site located on the globular domain of H1^17^. This model is supported by a quantitative analysis of H1 interactions with DNA that forms a four-way junction. Confirming previous qualitative observations^4^, we demonstrate that H1 binds hundred-fold tighter to four-way junctions than to linear DNA. This is the same –fold preference as demonstrated for nucleosomes over free DNA. Like nucleosomes, a four-way junction is characterized by the close juxtaposition and angled arrangement of two gyres of DNA (Fig. 6a).

While this manuscript was under review, a crystal structure of the globular domain of chicken H5 in complex with a 165 bp nucleosome was published^38^, demonstrating contacts with both linker arms, albeit with unequal contributions. Since chicken H5 and mouse H1^0^ are 88% identical, it was suggested that they bind in the same manner. This is in agreement with our finding that the globular domain of H1 (H1_1–96_) has a slight preference for nucleosomes with symmetrically extending linker DNA. However, the affinity of this H1 construct is over three orders of magnitude weaker than what is observed for full length H1. We conclude that the H1 C-terminal tail interacts mainly with one DNA linker arm.

The natural substrate for linker histones are not mono-nucleosomes, but nucleosomal arrays in which nucleosomes are connected by an average of −65 bp of linker DNA (in the presence of H1.0), resulting in a 212 bp nucleosomal repeat length^2^. Trinucleosomes are minimal models for such nucleosomal arrays. These substrates bind H1 with similar affinity as single mono-nucleosomes with two 30 bp linkers. We observe H1-induced compaction of trinucleosomes, and this effect is also observed with trinucleosome substrates that bind only one single H1. Thus, a single H1 is capable of organizing linker DNA into the characteristic stem structure observed earlier^12^,^39^. A careful analysis of stoichiometries of H1-trinucleosome complexes provides additional insight into the binding mode of H1 (Fig. 6b). Our data suggests that the H1-induced formation of a linker DNA stem and the ensuing compaction of trinucleosomal arrays precludes binding of a second linker histone, even though one of the connecting DNA lengths (2 × 30 bp) would be sufficient to accommodate two H1 molecules, and the second linker arm would be available in its entirety (Fig. 6b). This could be due to steric or electrostatic effects due to the close proximity of two CTDs, as indicated in our speculative model (Fig. 6b, middle panel).

The C-terminal positively charged domain of H1 makes important contributions to the compaction of chromatin fibers (reviewed in^40^). The functions of the H1.0 CTD are distributed between two distinct subdomains^25^,^33^ that become folded upon interaction with DNA^27^. Unlike a previous study^21^, we show that the CTD makes a profound contribution to the interaction with a mono-nucleosome, as its complete deletion reduces affinity by over two-thousand fold. The region of the CTD closer to the globular domain (amino acids 96–121) appears to be more important than the terminal 70 amino acids, despite the fact that charges are distributed evenly along the tail^33^. Since the CTD amino acid sequence varies among different H1 variants, our finding suggests that different H1 variants bind chromatin with different affinities, and this is something we are interested in following up on in the near future.

Our data suggest that the CTD is mainly responsible for interactions with linker DNA, as its contribution to binding affinity for nucleosomal substrates becomes less pronounced as linker length is reduced. For nucleosomes with very short asymmetric linker arms that do not bind full length H1 (A1/10), partial or even complete CTD deletion permits H1 binding with reasonable affinity. This suggests that the charge of the CTD might be inhibitory when the length of the linker DNA is limiting. Similar inverse correlations between the effects of tail deletion and DNA linker length have been observed previously, and were interpreted as an ordering of the disordered H1 CTD in full length H1 upon binding, which comes at an entropic cost^21^.

Surprisingly, deleting a portion of the H1 CTD (i.e. the H1_1–121_ construct) has little effect on the interaction with trinucleosomes, despite the 20–100 fold reduction in affinity for the equivalent single nucleosomes. This is consistent with published results reporting no major effects of partial CTD deletion on chromatin interactions and indeed on chromatin folding *in vitro*^12^,^33^. Our results imply that the binding mode of H1 (and the relative contribution of the CTD) may be different between mono-nucleosomes and nucleosomal arrays. This might be due to restrictions in linker DNA geometry, or due to inter-nucleosomal interactions in nucleosomal arrays compared to mono-nucleosomes.

The hypothesis that amino acid differences in the histone variant H2A.Z affect linker histone binding stems from the observation that the H2A C-terminal tail becomes more folded upon H1 interaction with the nucleosome, indicating close interactions^9^. Indeed, a chimeric version of H2A designed to mimic the H2A.Z C-terminal tail essentially prevents H1 binding. In contrast, we find that the incorporation of native H2A.Z into mono-nucleosomes and nucleosomal arrays has no effect on the affinity of H1; rather, H1 binds both substrates with near-identical sub-nanomolar affinity as the major-type counterparts. We believe that this discrepancy is the result of assay conditions in the previous study that promote aggregation, and due to the use of a chimeric H2A.Z ‘mimic’. In sum, our results emphasize the value of accurate solution-state characterization of chromatin complexes.

## Materials and Methods

### H1 purification and labeling

Three derivatives of mouse H1.0 were investigated here: full-length H1 (H1_FL_; residues 1–193) and two C-terminal tail deletions H1_1–121_ and H1_1–96_, previously referred as Δ72 and Δ97^23^,^33^. Each derivative contains a point mutation of serine 18 to cysteine (S18C) to allow for fluorescent labeling (previously referred to as S20C). Amino acid numbering for mouse H1.0 constructs does not include the first two amino acids (cloned in to allow a restriction site in the plasmid), as mass spectrometry results have shown that these two residues are cleaved in the final protein product (data not shown).

All three H1 derivatives were expressed and purified as previously described^23^, with the following modifications. After purification over a Sephadex column, H1 fractions were concentrated and dialyzed (or diluted) to 250 mM NaCl (20mM Tris-HCl pH 8.3, 1mM EDTA, 1mM DTT), and then applied to two- 5ml HiTrap SP columns (GE) and a 1 ml HiTrap Benzamidine (sepharose) FF column (GE) in tandem, using buffer A (20mM Tris-HCl pH 8.3, 1mM EDTA, 1mM DTT, 0 mM NaCl) and elution buffer B (20mM Tris-HCl pH 8.3, 1mM EDTA, 1mM DTT, 1M NaCl). Purification of the C-terminal tail deletion constructs requires all buffers to be at pH 8.0 for H1_1–121_ and at pH of 7.8 for H1_1–96_, while the H1_1-193_ buffers were at a pH of 8.3. All H1 derivatives were labeled with Oregon Green (Molecular Probes, O-6010), as previous described^23^, except that removal of excess fluorophore was achieved by purification over a Superdex S200 16/60 column. All purified and labeled H1 preparations were immediately flash frozen in presence of 20% glycerol in small aliquots, and stored at −80 °C (Supplementary Figure 4a, b).

### DNA purification and nucleosome reconstitution

All DNA sequences are based on the ‘601’ nucleosome positioning sequence^41^. Unlabeled S30/30, S17/13, A18/1 and S7/11 601 DNA constructs (see Fig. 1A for terminology), were purified as previously described^23^,^42^ with a final purification step over a MonoQ 10/100 column. LE, NLE, and NCP 601 sequences were purified as previously described, with the following variations: PEG 6000 concentration after EcoRV digestion was 5.4, 5.8 and 8.99%, respectively, and each sample was purified over a MonoQ 10/100 column. Sequences are shown in Supplementary Figure 4c.

Atto-647N labeled DNA fragments were synthesized using PCR from a pUC19 plasmid containing a single copy of the S30/30 601 sequence. Atto647N-NHS Ester (Sigma, 18373) was chemically attached to the reverse 30 bp primer which contained an internal amino modifier C6dT (shown in bold) 11 bp from the end of the sequence. All DNA generated by PCR was purified by Zymo DNA Clean & Concentrator kit and/or monoQ 10/100 column.

30 bp Rev primer 5′ATCATTAATA**T**GAATTCGCCACATGCA3′.

A labeled 30 bp fragment was prepared by annealing the 30 bp reverse primer (see above) to its reverse compliment at a 1:1 stoichiometry.

The A1/10 fragment was synthesized via PCR from the S7/11 template. The product was then purified over a monoQ 10/100 column. The purity of the A1/10 fragment (from the S7/11 template) was confirmed through AccI digestion analyzed on a 10% TBE gel.

Sequences and annealing of the 4 way T-junction DNA are as described^31^,^43^.

*Xenopus laevis* and *Mus musculus* core histones were purified, and nucleosomes were reconstituted for each of the different 601 fragments as described^42^. Trinucleosomes were reconstituted, and saturation verified by EcoRI digestion and AUC (Supplementary Figure 4d, e) following published protocols^44^,^45^,^46^.

### Fluorescence Resonance Energy Transfer (FRET) assays

Binding affinities were measured using the previously developed HI-FI FRET assay^23^ with Oregon Green 488 labeled H1 as the donor and nucleosomes (or DNA) labeled with Atto647N as the acceptor. The salt concentration was 150 mM KCl, unless stated otherwise. H1 was held constant and nucleosome (or free DNA) was titrated to a final concentration of 20 nM or 200 nM for H1_1–96_, unless otherwise indicated; 200 and 1000 nM was used for FRET with 30 bp free DNA. Each biological replicate was performed in duplicate. Optimal H1 concentration was determined by performing experiments with a range of 0.08–1 nM H1_FL_, which were then fit globally or individually with *Equation* 3. An H1_FL_ concentration of 0.5 nM was chosen for subsequent experiments, because it represented a consistent and robust signal change with affinities comparable with the globally fit data. FRET signal was background-subtracted and corrected for spectral overlap, as previously described^23^. The data was fit using Graphpad Prism, to the following equation representative of single-site specific binding:

SINGLE-SITE BINDING


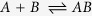


where,


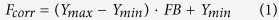


where Y_min_ and Y_max_ are the minimum and maximum F_corr_ signals, respectively. FB is defined as:


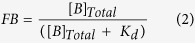


where A is the probe (typically H1), B is the titrant (typically the nucleosome), and Y_min_ and Y_max_ are the minimum and maximum F_corr_ signals, respectively. In the case where the apparent binding affinity was not more than two-fold below the concentration of A, we used the following quadratic equation, which incorporates the concentration of the probe:


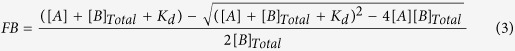


### Competition assays

Competitive binding experiments were performed using the HI-FI competition assay^23^. Briefly, H1 concentration was kept constant at 1 nM and pre-incubated with 10 nM (or 20 nM for H1_1–96_) S31/30 nucleosome (acceptor) at room temperature for ~15 minutes. Unlabeled competitor was then titrated and loss of F_corr_ quantified using ImageQuant, and then plotted in Graphpad Prism software. The IC_50_, or amount of competitor needed to compete 50% of the FRET interactions, was calculated as follows:


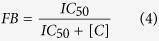


where C is the amount of unlabeled competitor added. Binding affinities (K_d_) were derived from the IC_50_ using the following equation:


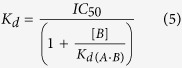


where B is the concentration of labeled ligand that is challenged by C, and A is the labeled substrate bound by B or C. A was typically held greater than 5-fold below the anticipated IC_50_ value, while B was typically held greater than 5-fold above the A-B affinity.

### (De)quenching

HI-FI (de)quenching assays were performed as described^23^. Briefly, labeled H1 was kept at a constant concentration of 0.08–0.1 nM (unless otherwise stated) and unlabeled nucleosome was titrated. The fluorescent signal was quantified using ImageQuant and then plotted in Graphpad PRISM and fit to the single site binding (*Eq.* 2) or quadratic equation (*Eq.* 3) above.

HI-FI (de)quenching stoichiometry measurements were performed with labeled H1_FL_ at a constant concentration of 10 nM; unlabeled trinucleosome was titrated from 0.8–40 nM. The fluorescent signal was quantified using ImageQuant and then plotted in Graphpad PRISM to fit straight lines.

### Native PAGE

EMSA were performed in a 5% native PAGE gel at 0.2X TBE with labeled H1 and titrated nucleosomes (labeled for FRET and unlabeled for competition) as previously described^23^. Gels were run at 300V for 3 hrs at 4 °C and then scanned on Typhoon imager using the indicated channels.

### Atomic Force Microscopy

NLE-Tri without and with H1 were imaged as described^46^ with the following exceptions. NLE-Tri was diluted in TCS buffer (20 mM Tris pH7.5, 1 mM EDTA, and residual DTT) to a final concentration of 3.135 nM (1.16 ng/μl). 30–45 μl of this concentration (3.135 nM) was placed on a mica slide and incubated for 5–15 minutes, rinsed in 500 μL TCS, dried and then imaged on an Asylum Research MFP-3D Atomic Force Microscope. Images were collected at 1 × 1 μm −500 × 500 nm scans and digitally zoomed. NLE-Tri/H1 complex was prepared in the following manner. NLE-Tri and H1 were diluted in TCS and mixed at a 1 NLE-Tri to 1 H1 ratio at a concentration of 5.23 nM and incubated at RT for ~20 mins. The NLE-Tri/H1 complex was then diluted in TCS to a final concentration of 2.615 nM (~1.16 ng/μl), 40 μl was added to the mica slide and imaged as described above. Height traces were completed using the MFP-3D software on a minimum of nine different images for a total of 1005 particles.

Geometry was determined by digital zoom of a minimum of seven separate images and only those particles in which three nucleosomes could be seen were counted. Particles over 4 nm were likewise excluded. The distance between the nucleosomes was determined by indicated scale on individual images. Particles were grouped together into either an open or a condensed geometry (Fig. 4c). Particles with measured heights of 2.5–4 nm (where individual nucleosomes could not be distinguished) were also binned.

## Additional Information

**How to cite this article**: White, A. E. *et al.* A quantitative investigation of linker histone interactions with nucleosomes and chromatin. *Sci. Rep.* **6**, 19122; doi: 10.1038/srep19122 (2016).

## Supplementary Material

Supplementary Information

## Figures and Tables

**Table 1:** Summary of Binding Affinities.

Substrate	H1_1-193_	Error	H1_1–121_	error	H1_1–96_	error
	K_d_ [nM]		K_d_ [nM]		K_d_ [nM]	
DNA*	**7.000**	+1.930 − 1.51 (n = 4)	**69.677**	+72.60 − 35.6 (n = 3)	**1446.503**	+131.7 − 120.7 (n = 3)
S31/30*	**0.0484**	+0.027 − 0.017 (n = 4)	**0.983**	+0.229 − 0.186 (n = 5)	**86.267**	+9.087 − 8.22 (n = 2)
S30/30	**0.0177**	+0.004 − 0.003 (n = 7)	**1.850**	+0.282 − 0.244 (n = 3)	**123.626**	+14.30 − 12.82 (n = 2)
A3/30*	**0.1120**	+0.066 − 0.046 (n = 2)	**10.428**	+2.655 − 2.116 (n = 3)	**247.517**	+36.52 − 31.82 (n = 3)
S17/13	**0.2301**	+0.032 − 0.028 (n = 3)	**0.947**	+0.095 − 0.086 (n = 3)	**26.507**	+2.41 − 2.21 (n = 3)
A18/1	**0.1352**	+0.410 − 0.32 (n = 5)	**8.082**	+0.725 − 0.665 (n = 3)	**208.144**	+78.10 − 78.1 (n = 2)
S7/11	**1.7309**	+0.221 − 0.196 (n = 3)	**3.256**	+0.330 − 0.300 (n = 3)	**73.622**	+17.3 − 17.3 (n = 2)
A1/10	**NC**	NA	**43.810**	+0.247 − 0.246 (n = 2)	**139.853**	+17.31 − 15.4 (n = 2)
NCP	**NC**	NA	**NC**	NA	**NC**	NA
S30/30-H2A.Z^◊^	**0.051**	+0.0325 − 0.0198 (n = 3)	**ND**	NA	**ND**	NA
NLE-TRI^◊^	**0.042**	+0.025 − 0.015 (n = 4)	**0.0638**	+0.009 − 0.001 (n = 2)	ND	NA
LE-TRI^◊^	**0.046**	+0.032 − 0.0191 (n = 6)	**0.047**	+0.027 − 0.017 (n = 2)	ND	NA
NLE-Tri-H2A.Z^◊^	**0.023**	+0.0118 − 0.0078 (n = 2)	**ND**	NA	ND	NA
S30/30^◊^	**0.039**	+0.0064 − 0.0055 (n = 8)	ND	NA	ND	NA
4WJ15^+◊^	**0.035**	+0.0011 − 0.0017 (n = 2)	ND	NA	ND	NA
4WJDNA^+◊^	**3.750**	+0.450 − 0.41 (n = 2)	ND	NA	ND	NA

Upper and lower error for each value is listed, together with the number of independent replicates (n). NC is no change in fluorescent signal; NA stands for ‘not applicable’; ND is ‘not determined’. Values measured by direct FRET are indicated (*); while (^◊^) denotes values determined by fluorescence (de)quenching. All other values were obtained from competition experiments. (+) denotes measurement performed at 20 mM KCl. For measurements with two replicates, the errors represent the upper and lower K_d_’s. All other errors are based on a 95% confidence interval, derived by taking the log of the K_d_. All substrates are shown schematically in Figs 1a and 3a.
