# Supplementary material for: A quantitative investigation of linker histone interactions with nucleosomes and chromatin
Source: Sci Rep. 2016 Jan 11;6:19122. doi: 10.1038/srep19122 (PMC4707517; doi:10.1038/srep19122)
Supplement: Supplementary Information [file srep19122-s1.pdf]

A quantitative investigation of linker histone interactions with nucleosomes and chromatin

[1]Alison E. White, Aaron R. Hieb, and [1]Karolin Luger

Supplementary Figures:

Supplementary Figure 1: Validation of FRET and competition assays

a) 5% native PAGE of samples taken from a HI-FI competition experiment between S31/30 (10 nM) and A1/10 (0-500 nM), for $H1_{FL}$ (1 nM). The gel was visualized at the indicated wavelengths, then stained with ethidium bromide. Lanes 1-6 are $H1_{FL}$ with decreasing amounts of A1/10 (500, 62.5, 15.6, 1.95, 0.244, 0.0305 nM, respectively). Lane 7 is S31/30 Atto647N nucleosome alone. Donor signal with $H1_{FL}$ remains with S31/30 nucleosome as more A1/10 nucleosome competitor is added (lanes 1-6: middle gel) indicating that A1/10 nucleosome is unable to compete $H1_{FL}$ from the S31/30 nucleosome.

b) Representative (de)quenching curves of S30/30 nucleosome with the H2A.Z histone variant (S30/30.z) reconstituted with mouse histones, to measure the interaction with $H1*_{FL}$. $H1_{FL}$ was held constant at 0.08-0.1 nM and S30/30.z nucleosome was titrated (0-25 nM). Curves were fit with a quadratic equation (*Eq. 3*).

c) Representative (de)quenching curve of NLE-Tri (NLE-Tri.z) nucleosome with the H2A.z histone variant containing mouse histones, upon binding to $H1*_{FL}$. $H1_{FL}$ was held constant at 0.08-0.1 nM and NLE-Tri.z nucleosome was titrated (0-25 nM). Curves were fit with *Eq. 3*.

Supplementary Figure 2: Analysis of NLE-Tri – H1 complexes by Atomic Force Microscopy

NLE-Tri was imaged with AFM alone or in presence of $H1_{FL}$ (molar ratio of 1 H1 per 1 NLE-Tri array).

a) Digital zooms of AFM scan with example height trace(s) of NLE-Tri alone (left) or with $H1_{FL}$ (right). Height increases 1.3 to 1.9 nm when $H1_{FL}$ is present.

b) Upper two panels: Digital zoom of scans of NLE-Tri alone showing the open geometry of the trinucleosome. Lower two panels: Digital zoom of scans of NLE-Tri in the presence of $H1_{FL}$ depicting the closed trinucleosome.

Supplemental Figure 3: Representative FRET curves, and competition with H1 C-terminal tail deletion constructs.

a) Representative competition experiment between S31/30 (10 nM) and the indicated unlabeled nucleosome (0-500 nM) for $H1_{1\text{-}121}$ (1 nM). Curves were fit with *Eq. 4.*

b) Representative competition curves between S31/30 (20 nM) and the indicated unlabeled nucleosome (0-500 nM) for $H1_{1\text{-}96}$ (1 nM). Curves were fit with *Eq. 4.*

c and d) Representative (de)quenching isotherm of (c) LE-Tri (0-25 nM) or (d) NLE-Tri (0-25 nM) for $H1_{1\text{-}121}$ (0.1 nM). Data were fit with *Eq 3*.

Supplemental Figure 4: Validation of NLE-Tri and LE-Tri saturation and H1 purification.

a) Representative (de)quenching curves of $H1_{FL}$ purified with the published method demonstrating the effect of prep age on $H1_{FL}$ affinity. Upper panel: bi-phasic nature of H1-nucleosome interaction after storage of H1 at 4^0C for ~4 days. The gray curve is S30/30 (from figure 3b right). Lower panel: the bi-phasic curve (above) separated into 2 binding isotherms (lower and upper) fit with *eq 3. Lower portion (black) has a* K_d *of 0.022* $^+/_-$ *0.0046 (*R^2*=0.901); upper portion (dark gray) has a* K_d *of 3.32* $^+/_-$ *0.87 (*R^2*=0.89).*

b) 15% polyacrylamide SDS PAGE of H1 derivatives using an improved purification method (Lanes 2-6), fluorescent image (top) and Imperial protein stain (bottom). Lanes 2-4 are the indicated H1 derivative which had previously been frozen. Lane 5: freshly made protein; lane 6: unlabeled $H1_{FL}$. Degradation of $H1_{FL}$ occurs rapidly (in less than one week) at 4^0C storage (lane 7); this is only seen when visualized by fluorescence.

c) Sequences of all mono-nucleosome DNA fragments used in this study. Trinucleosomes sequences are 3 copies of S30/30.

d) Trinucleosomes were analyzed for degree of saturation. EcoRI digestion of NLE-Tri (top) and LE-Tri (bottom); the absence of free 207 DNA indicates the trinucleosome is saturated. U: uncut, C: cut. Lane 1: uncut trinucleosome; lane 2: EcoRI-treated; lane 3: S30/30 nucleosome control; lane 4: S30/30 bp DNA.

e) Analysis of trinucleosomes by analytical ultracentrifugation (AUC). Sedimentation coefficients ($S_{(20,w)}$) for trinucleosome substrates. NLE-Tri = ~16S; LE-Tri = ~18S. Both trinucleosome

substrates were reconstituted with mouse histones and have a slightly different S_{50} compared to published results using *Xenopus laevis* histones [1].

References

1 Winkler, D. D., Luger, K. & Hieb, A. R. Quantifying Chromatin-Associated Interactions: The HI-FI System. *Methods Enzymol* **512**, 243-274, doi:B978-0-12-391940-3.00011-1 [pii] 10.1016/B978-0-12-391940-3.00011-1 (2012).

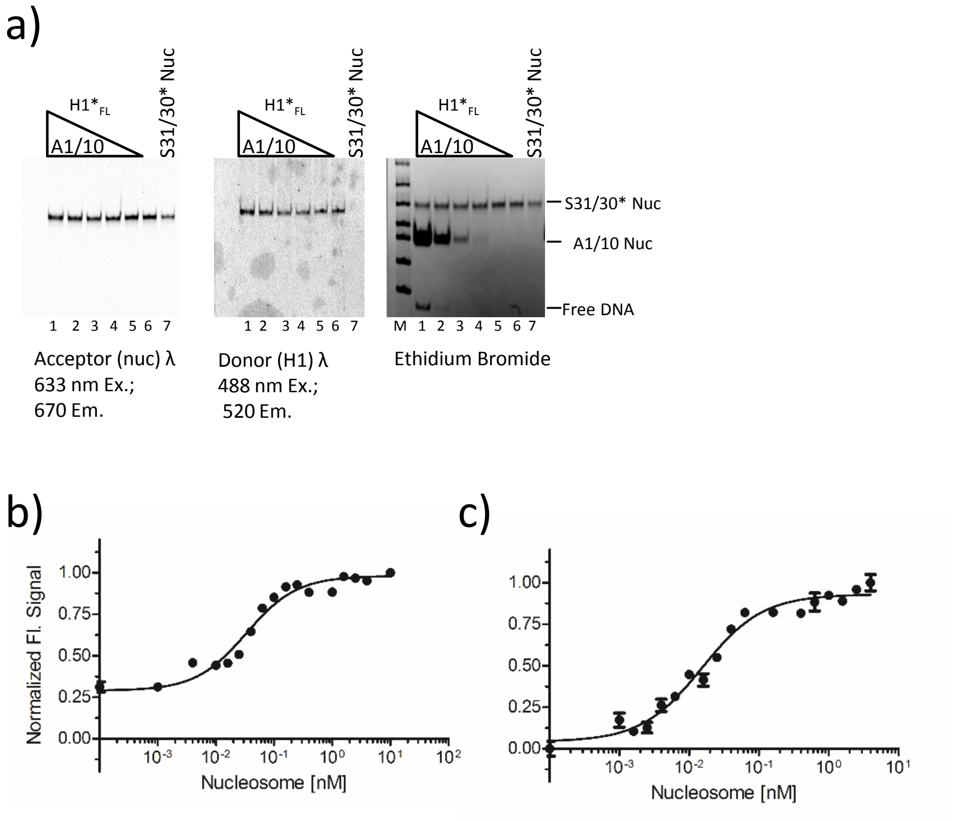

Supplementary Figure 1

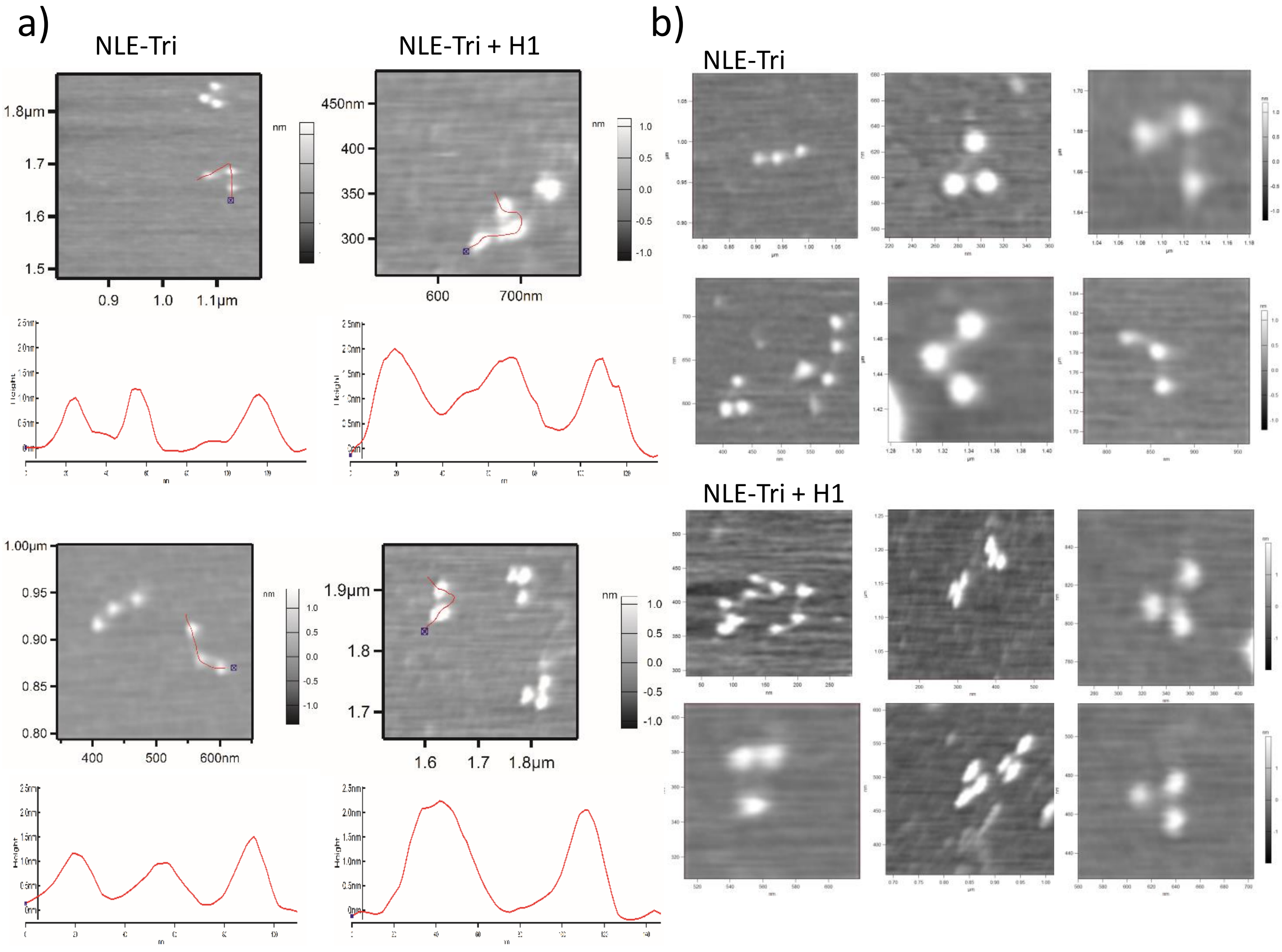

Supplementary Figure 2

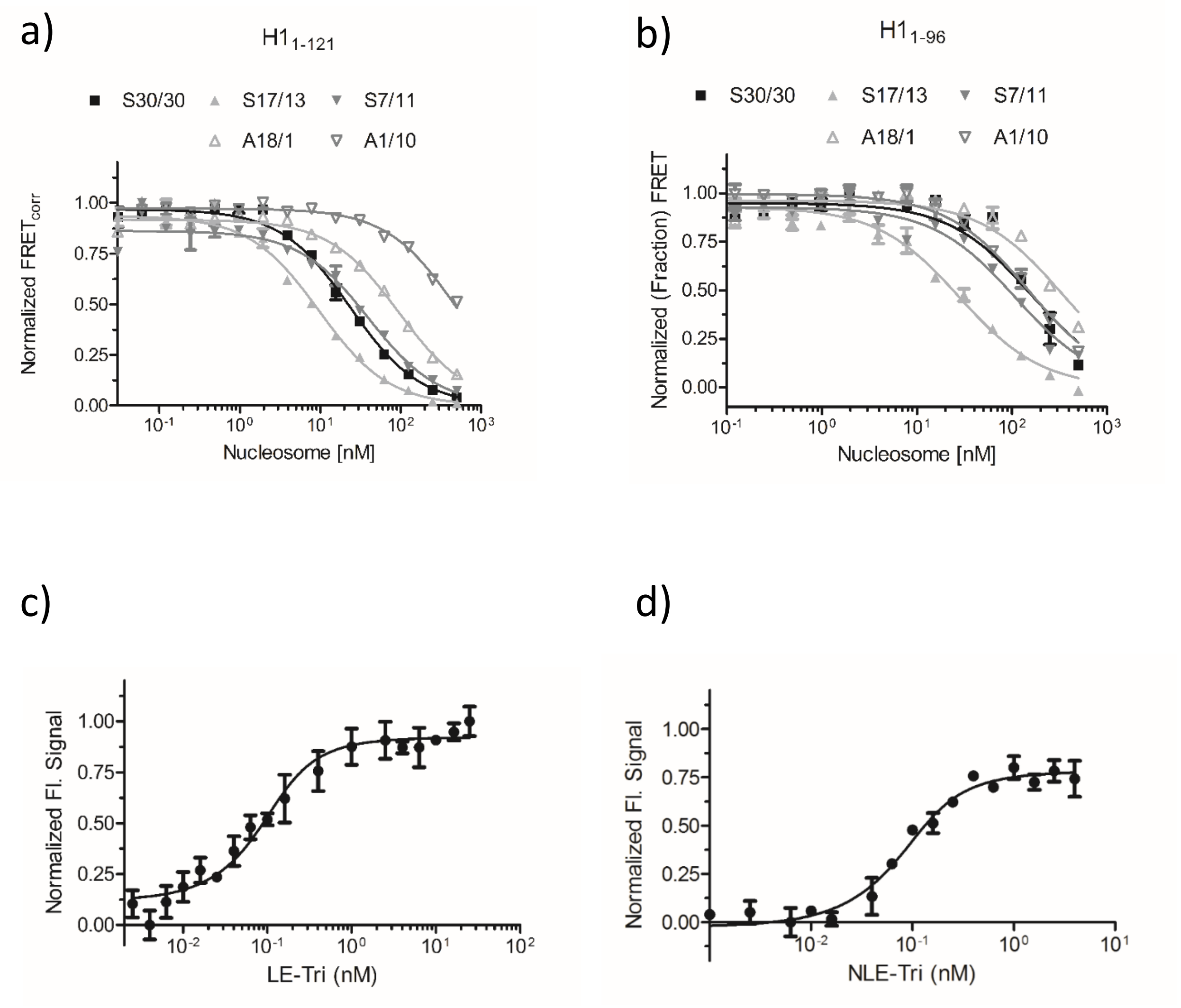

Supplementary Figure 3

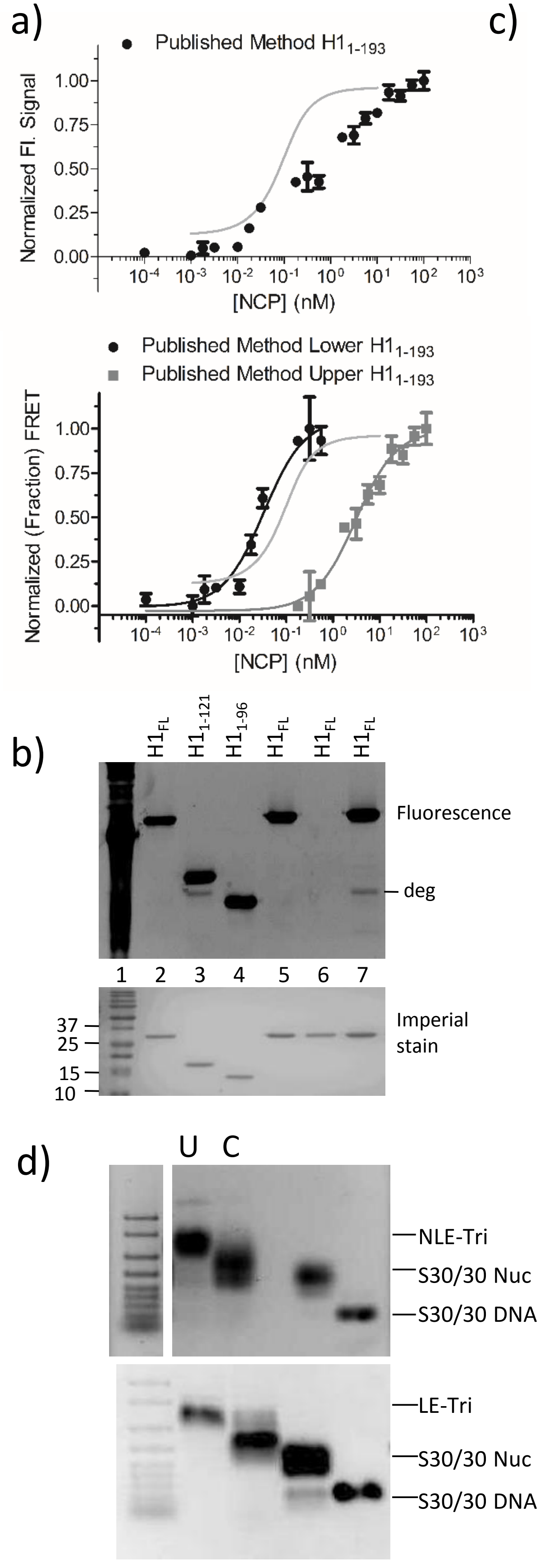

c)

```
S30/30       -ATCTAATACTAGGACCCTATACGCGGCCGCATCGGAGAATCCCGGTGCC
S31/30*      GATCTAATACTAGGACCCTATACGCGGCCGCATCGGAGAATCCCGGTGCC
A3/30*       ----------------------------CGCATCGGAGAATCCCGGTGCC
S17/13       --TC--------G----GGATACGCGGCCGCCCTGGAGAATCCCGGTGCC
A18/1        -ATC--------C----CTATACGCGGCCGCCCTGGAGAATCCCGGTGCC
S7/11        ---C--------G-----------AGCCAG-GCCTGAGAATC-CGGTGCC
A1/10        ------------------------A----G-GCCTGAGAATC-CGGTGCC
NCP          -----------------------------G-GCCTGAGAATC-CGGTGCC
                                          *     ******* *******

S30/30       GAGGCCGCTCAATTGGTCGTAGACAGCTCTAGCACCGCTTAAACGCACGT
S31/30*      GAGGCCGCTCAATTGGTCGTAGACAGCTCTAGCACCGCTTAAACGCACGT
A3/30*       GAGGCCGCTCAATTGGTCGTAGACAGCTCTAGCACCGCTTAAACGCACGT
S17/13       GAGGCCGCTCAATTGGTCGTAGACAGCTCTAGCACCGCTTAAACGCACGT
A18/1        GAGGCCGCTCAATTGGTCGTAGACAGCTCTAGCACCGCTTAAACGCACGT
S7/11        GAGGCCGCTCAATTGGTCGTAGACAGCTCTAGCACCGCTTAAACGCACGT
A1/10        GAGGCCGCTCAATTGGTCGTAGACAGCTCTAGCACCGCTTAAACGCACGT
NCP          GAGGCCGCTCAATTGGTCGTAGACAGCTCTAGCACCGCTTAAACGCACGT
             **************************************************

S30/30       ACGCGCTGTCCCCCGCGTTTTAACCGCCAAGGGGATTACTCCCTAGTCTC
S31/30*      ACGCGCTGTCCCCCGCGTTTTAACCGCCAAGGGGATTACTCCCTAGTCTC
A3/30*       ACGCGCTGTCCCCCGCGTTTTAACCGCCAAGGGGATTACTCCCTAGTCTC
S17/13       ACGCGCTGTCCCCCGCGTTTTAACCGCCAAGGGGATTACTCCCTAGTCTC
A18/1        ACGCGCTGTCCCCCGCGTTTTAACCGCCAAGGGGATTACTCCCTAGTCTC
S7/11        ACGCGCTGTCCCCCGCGTTTTAACCGCCAAGGGGATTACTCCCTAGTCTC
A1/10        ACGCGCTGTCCCCCGCGTTTTAACCGCCAAGGGGATTACTCCCTAGTCTC
NCP          ACGCGCTGTCCCCCGCGTTTTAACCGCCAAGGGGATTACTCCCTAGTCTC
             **************************************************

S30/30       CAGGCACGTGTCAGATATATACATCGATTGCATGTGGATCCGAATTCATA
S31/30*      CAGGCACGTGTCAGATATATACATCGATTGCATGTGGATCCGAATTCATA
A3/30*       CAGGCACGTGTCAGATATATACATCGATTGCATGTGGATCCGAATTCATA
S17/13       CAGGCACGTGTCAGATATATACATCCTGTGCATGTATTGAC---------
A18/1        CAGGCACGTGTCAGATATATACATCCGAT---------------------
S7/11        CAGGCACGTGTCAGATATATACATCCAGGCCTTGTGTCG-----------
A1/10        CAGGCACGTGTCAGATATATACATCCAGGCCTTGTGTC------------
NCP          CAGGCACGTGTCAGATATATACATCCAG----------------------
             *************************

S30/30       TTAATG-AT 207
S31/30*      TTAATG-AT 208
A3/30*       TTAATG-AT 180
S17/13       --------- 177
A18/1        --------- 166
S7/11        --------- 165
A1/10        --------- 158
NCP          --------- 147
```

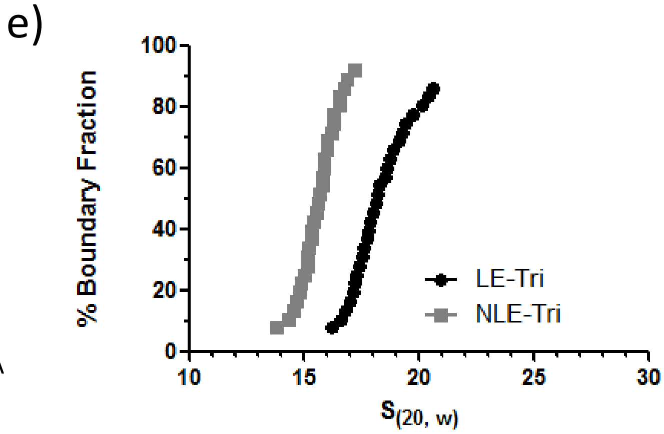

Supplementary Figure 4
